# Impact of physical and mental health on life satisfaction in old age: a population based observational study

**DOI:** 10.1186/s12877-016-0365-4

**Published:** 2016-11-25

**Authors:** Thomas Puvill, Jolanda Lindenberg, Antonius J. M. de Craen, Joris P. J. Slaets, Rudi G. J. Westendorp

**Affiliations:** 1 Leyden Academy on Vitality and Ageing, Rijnsburgerweg 10, 2333 AA Leiden, The Netherlands; 2 Department of Gerontology and Geriatrics, Leiden University Medical Center, Albinusdreef 2, 2333 ZA Leiden, The Netherlands; 3 University of Groningen, University Medical Center Groningen, Hanzeplein 1, 9713 GZ Groningen, The Netherlands; 4 Department of Public Health and Center of Healthy Ageing, Copenhagen University, Øster Farimagsgade 5, Postboks 2099, 1014 København K, Denmark

**Keywords:** Life satisfaction, Physical health, Mental health, Vitality, Cohort study

## Abstract

**Background:**

It is widely assumed that poor health lowers life satisfaction when ageing. Yet, research suggests this relationship is not straightforward. This study investigated how older people evaluate their life when facing disease and disabilities.

**Methods:**

The Leiden 85-plus Study, a prospectively followed cohort of a cohort of a middle-sized city in the Netherlands, all aged 85 years, that was age-representative of the general population, was used. Those with severe cognitive dysfunction were excluded (*n* = 501). Comorbidities, physical performance, cognitive function, functional status, residual lifespan, depressive symptoms and experienced loneliness were measured during home visits. Life satisfaction was self-reported with Cantril’s ladder. All analyses were performed using regression analysis.

**Results:**

Participants reported high life satisfaction (median 8 out of 10 points) despite having representative levels of disease and disability. Comorbidity, low cognitive function, and residual lifespan as markers of health were not associated with life satisfaction. Poor physical performance and low functional status were weakly but significantly associated with lower life satisfaction (*p* < 0.05 respectively *p* < 0.001), but significance was lost after adjustment for depressive symptoms and perceived loneliness. Depressive symptoms and perceived loneliness were strongly related to lower life satisfaction (both *p* < 0.001), even after adjustment for physical health characteristics.

**Conclusion:**

Poor physical health was hardly related to lower life satisfaction, whereas poor mental health was strongly related to lower life satisfaction. This indicates that mental health has a greater impact on life satisfaction at old age than physical health, and that physical health is less relevant for a satisfactory old age.

## Background

Advanced age is no longer for the lucky few. It is expected that by 2050, one out of four people will be 60 years or older, with the age group of over 80 experiencing the most rapid relative increase [1]. Unfortunately, the years that we have added to our lifespan are not free of disease and disability [2, 3]. The prospect of decline in bodily functions often disconcerts younger and older individuals: it is widely assumed, and some evidence suggests [4], that a good physical health is imperative for life satisfaction in old age.

Discordant with these assumptions, life evaluations have been found to be largely stable across the lifespan and not parallel to trajectories of physical decline [5, 6]. This stability of life satisfaction far into old age has been coined the ageing paradox, and can be observed until around age 70 when some studies find that life satisfaction starts to decline [7, 8]. Even at this age, however, the life satisfaction of many individuals remains high in spite of physical decline [6, 9]. This raises the question for whom life satisfaction does and does not decrease. It has even led some authors to argue that life satisfaction and physical decline are not directly related at all [10]. The answer is of importance not only for older people but also to healthcare professionals and policy makers, who aim to optimise both physical functioning and life satisfaction.

The objective of this study was to uncover the impact of physical and mental health on life satisfaction at advanced age. To this end, we analysed data from the Leiden 85-plus study, a representative population-based cohort of 85-year-old citizens.

## Methods

### Design and study sample

For the current paper, we used data from the first wave of the Leiden 85-plus Study. All inhabitants of the Dutch municipality of Leiden who turned 85 between 1 September 1997 and 1 September 1999 (*n* = 705) were identified from the municipal’s register. No exclusion criteria were employed at inclusion, but for the current analysis, participants were excluded if they had severe cognitive dysfunction, operationalized as MMSE score < 19 points, as self-reported questionnaires on mental health characteristics were not administered to these subjects [11]. Out of the eligible population, 14 died before enrolment, and 92 declined participation. The remaining 599 participants (87%) agreed to participate [11]. 98 Participants had an MMSE score below 19 points, setting the final study sample at 501.

### Measures

Participants received home visits of a trained researcher three or four times in their 85^th^ year during which standardised interviews and physiological measurements were conducted [11, 12]. All measurements were performed only once per year. Included measures are hereafter divided in demographics, physical health, mental health characteristics, and life satisfaction.

Data on demographics, sex and marital status was collected from the municipality. Participants provided information on their income (state pension or state pension and additional income), education (primary school or primary school and additional education) and living situation (independent or institutionalized) during one of the interviews.

Physical health was determined as presence of chronic diseases, physical performance, functional status, cognitive function, and residual lifespan. Number of diseases were counted as described previously [12]. Level of physical performance was based on outcomes for upper body physical performance by handgrip strength and lower body physical performance by gait speed [12]. For estimating handgrip strength, participants were asked to grip a dynamometer as hard as they could three times, and their best attempt was used as an indication of their handgrip strength. For gait speed, participants were asked to walk a short distance, using walking aids if necessary. Gait speed was based on the first three meters of the task. Scores for both these measures were ranked in quintiles for males and females separately to account for men’s generally higher level of performance. Quintile scores for upper and lower body physical performance were added up for the final score. Functional status was based on activities of daily living (ADL) and instrumental activities of daily living (IADL) as self-reported on the Groningen Activity Restriction Scale [13]. ADL and IADL scores were combined into a total score ranging from 18 to 72, so that lower numbers indicate more dysfunction. To facilitate interpretation, the aggregated score was coded in reverse for analysis. Cognitive function was measured with the Mini-Mental State Examination (MMSE) [14], a screening instrument for cognitive dysfunction. Scores range from 0 to 30, higher scores indicating better cognitive function. Information on residual lifespan – the number of years the participant lived after age 85 - was obtained from the municipal registry. For analysis, the total disease count was categorised as ‘comorbidity’ (multiple chronic diseases) or less than two diseases. For figures, categories 0, 1, 2 and 3+ diseases were used. Due to lack of a (consensual) cut-off score in the literature, all other physical health variables were categorised above or below the median for analysis and in quartiles for figures, to allow effect sizes to be compared (see Table 1 for the dichotomous cut-off scores).

**Table 1:** Characteristics of the participants at age 85

Characteristics	*N* = 501
Demographics	
Female / male	317 (63.3%) / 184 (36.7%)
Married / not married	176 (35.1%) / 325 (64.9%)
State pension / state pension and extra income	71 (14.1%) / 430 (85.9%)
Only primary school / further education	304 (60.7%) / 197 (39.3%)
Institutionalised / living independently	55 (11.0%) / 446 (89.0%)
Physical health	
None or one disease / more than one disease	376 (75.0%) / 125 (25.0%)
Grip strength in kilograms (median, IQR)	22 (18–29)
Gait speed in seconds (median, IQR)	4.0 (3.1–5.8)
Cognitive function score ≤ 26 / ≥ 27^a^	232 (46.3%) / 269 (53.7%)
Functional status score ≤ 26 / ≥ 27^a^	238 (47.5%) / 263 (52.5%)
Residual lifespan ≤ 5 / ≥ 6 years^a^	256 (51.1%) / 245 (48.9%)
Mental health	
Depressive symptoms score ≤ 4 / ≥ 5	78 (15.6%) / 423 (84.4%)
Perceived loneliness score ≤ 2 / ≥ 3	128 (25.5%) / 373 (74.5%)
Life satisfaction	
Life satisfaction score (median, IQR)	8 (7–9)
Life satisfaction score ≤ 5	59 (11.8%)
Life satisfaction score 6	58 (11.6%)
Life satisfaction score 7	105 (21%)
Life satisfaction 8	143 (28.5%)
Life satisfaction score 9	48 (9.6%)
Life satisfaction score 10	88 (17.6%)

Data are number of patients (%) or median (IQR) For detailed descriptions of the variables, see Methods

^a^The cut-off was based on the median

For mental health characteristics, depressive symptoms were measured with the Geriatric Depression Scale 15 (GDS-15) [12]: a 15-item screening instrument for depression developed for older populations. For analysis, the outcome score that can range from 1 to 15 was divided into score ≤ 4 for less depressive symptoms and ≥ 5 for more depressive symptoms [15] and for figures, the outcomes were divided into scores ≤ 4, 5–9, and ≥ 10 [16]. Perceived loneliness was measured with the De Jong-Gierveld Loneliness scale [12]: an 11-item questionnaire designed to measure loneliness with scoring range 0 to 11. For analysis, the total scores were categorized into ≤ 2 for those who did not experience loneliness and ≥ 3 for those who did, and for figures scores were categorized into ≤ 2, 3–9, and ≥ 10 [17].

Life satisfaction was measured with Cantril’s 10-point self-anchoring ladder [18]. Participants were presented with a depiction of a ladder, the steps numbered one to ten. Participants were told that the bottom step is the worst and the top step is the best imaginable life for them and were asked to indicate where on the ladder they felt they stood at that time, resulting in a life satisfaction score ranging from 1 to 10.

### Data analysis

#### Main analyses

To identify determinants of life satisfaction, three sets of multiple linear regression analyses were performed with SPSS Statistics for Windows (IBM, Armonk, NY). The first of the three sets consisted of regression analyses for each physical and mental health characteristic separately, correcting for demographic characteristics only. The second set consisted of regression analyses for each physical health characteristic, correcting for demographic and all mental health characteristics. The third set consisted of regression analyses for each mental health characteristic, correcting for demographic and all physical health characteristics.

#### Categorisation

In all analyses, predictor variables were added simultaneously to the model as binary variables. Dichotomisation was done as described under ‘Measures’. Figures were supplemented with trend analyses using uncategorised, full-range variables.

#### Effect sizes

Coefficients of determination (percentage of explained variance) were calculated in order to estimate the effect size of the association between life satisfaction and characteristics of physical and mental health. For this, the full-range variabl/es were used.

#### Heteroscedasticity

To ensure that our results were not influenced by heteroscedasticity, we repeated the analyses using heteroscedasticity-consistent standard errors (HCSE) [19]. The results were not substantially different; therefore we here provide the outcomes of the regular analyses.

#### Missing values

In total 1.22% of all data was missing. Missing values were imputed with multiple imputation using all full-range variables in this study as predictors. A sensitivity analysis was performed excluding the imputed data. This gave a similar pattern of results.

#### Ethics, consent and permission

The study was approved by the Medical Ethical Committee of the Leiden University Medical Center. Permission to participate was provided in writing by participants who were able to do so. For cognitively impaired individuals, permission to participate was provided in writing by a guardian.

## Results

### Description of participants

The original sample of the Leiden 85-plus Study included 599 individuals and was representative of the entire source population that was intended for recruitment and the general Dutch population in terms of physical health, mortality hazard and demographics [11, 20]. For present analyses we excluded 98 participants with severe cognitive impairment, which was operationalized as an MMSE score below 19 points. Table 1 shows characteristics of the 501 participants included in the analyses. Life satisfaction was rated highly by most participants (median 8 out of 10 points (IQR 7 to 9)). Note the additional peak in the distribution at score 10 points (*n* = 88). Six participants rated their life satisfaction with 1 point and 21 participants rated it with less than 5 points. Ratings of life satisfaction were available for 43 of the 98 participants who were excluded due to severe cognitive impairment; the median score in this group was 8 out of 10 points (IQR 6 to 8).

### Determinants of life satisfaction

There were no differences in life satisfaction between the sexes (0.1 points, 95% CI −0.4 to 0.3), nor between types of marital status (0.0 points, 95% CI −0.4 to 0.4), nor between those who had a state pension and those with additional income (0.0 points, 95% CI −0.4 to 0.5), nor between those who went to primary school and those with additional education (0.1 points, 95% CI −0.2 to 0.4). Life satisfaction among those who were institutionalized was 1.0 point lower (*p* < 0.001, 95% CI −1.5 to −0.5) than among those who were not institutionalized. This difference became smaller and lost statistical significance after adjustment for mental health characteristics (−0.5 points, 95% CI −1.0 to 0.1).

Table 2 shows the results of the regression analyses after dichotomising the various characteristics of health to enable the comparison of the various effect sizes. When adjusting for demographic characteristics only, the only physical health characteristics associated with lower life satisfaction were poorer physical performance and functional status (*p* < 0.05 and *p* < 0.001 respectively, both half a point). The negative association with physical performance was lost when further adjusting for mental health characteristics (0.2 points, 95% CI −0.1 to 0.5). The association with functional status weakened when further adjusting for mental health characteristics (*p* < 0.05, −0.3 points, 95% CI −0.6 to −0.1). In contrast to physical health characteristics, markers of poor mental health were strongly associated with lower life satisfaction (*p* < 0.001, one to two points) with and without adjustment for unequal distributions in physical health (Table 2).

**Table 2:** Self-perceived life satisfaction at age 85 by physical and mental health characteristics

	Difference in life satisfaction score between groups (points; 95% CI)		
Characteristics	Crude	Adjusted for mental health	Adjusted for physical health
Physical health			
Comorbidities vs less than two diseases	−0.1 (−0.5–0.2)	0.1 (−0.2 −0.4)	–
Below vs above median physical performance	−0.5 (−0.8–−0.2)*	0.2 (−1–0.5)	–
Below vs above median cognitive function	−0.3 (−0.6–0.0)	−0.1 (−0.4–0.2)	–
Below vs above median functional status	−0.5 (−0.9–−0.3)***	−0.3 (−0.6–0.1)*	–
Below vs above median residual lifespan	−0.2 (−0.5–0.2)	−0.0 (−0.3–0.3)	
Mental health			
Below vs above cut-off depressive symptoms	−2.1 (−2.5–−1.7)***	–	−2.0 (−2.4–−1.6)***
Below vs above cut-off perceived loneliness	−1.2 (−1.5–−0.8)***	–	−1.1 (−1.5–−0.8)***

All estimates from regression adjusted for sex, marital status, income, education and institutionalisation

Each of the estimates comes from a separate analysis

*p < ·05, ***p < ·001

**Fig. 1:**
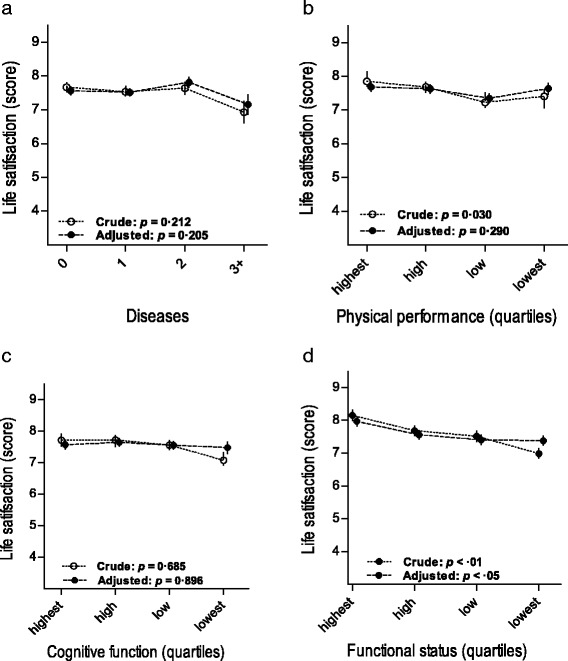
Life satisfaction at age 85 determined by physical health characteristics. Estimates represent marginal estimated mean scores; error bars represent standard errors of mean. Crude estimates were adjusted for demographic variables only and adjusted estimates for demographic and mental health characteristics. **a** Diseases. **b** Physical performance (quartiles). **c** Cognitive function (quartiles). **d** Functional status (quartiles)

**Fig 2:**
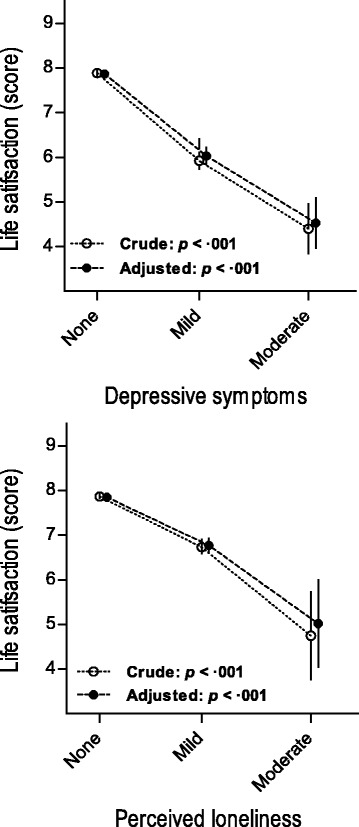
Life satisfaction at age 85 estimated by mental health characteristics. Estimates represent marginal estimated mean scores; error bars represent standard errors of mean. Crude estimates were adjusted for demographic characteristics only and adjusted estimates for both demographic and physical health characteristics

**Fig. 3:**
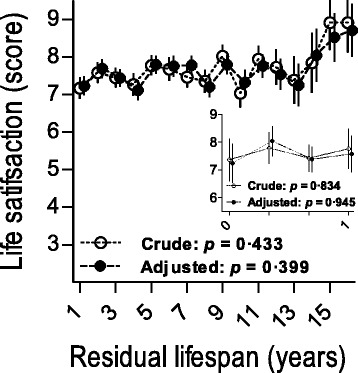
Life satisfaction estimated by residual lifespan, for all and for residual lifespan < 1 (*n* = 38). Estimates represent marginal estimated mean scores; error bars represent standard errors of mean. Crude estimates were adjusted for demographic variables only and adjusted estimates for demographic and mental health characteristics

To provide further information, we performed various trend analyses (Figs. 1, 2 and 3). Figure 1 shows mean life satisfaction estimated by various characteristics of physical health: comorbidity, physical performance score, cognitive function, and functional status. Trend analysis showed that poorer physical performance had a significant but small association with lower life satisfaction in the crude model (Fig. 1b), but lost significance after adjustment for characteristics of mental health. Poorer functional status showed a significant but small association with lower life satisfaction in both the crude and the adjusted model corrected for unequal distributions in mental health characteristics. Other physical health characteristics were not related to life satisfaction.

Figure 2 shows the mean life satisfaction dependent on characteristics of mental health: depressive symptoms and perceived loneliness. Trend analysis showed that having more depressive symptoms and perceiving greater loneliness were strongly and significantly associated with lower life satisfaction (both *p* < 0.001). Further adjustment for unequal distributions of physical health did not substantially change these results.

All participants were followed up for vital status. Figure 3 shows ratings of life satisfaction at age 85 years dependent on their residual lifespan. No significant relation was observed, neither for those who died within one year after assessment, nor for those who died later in time. Participants who lived up to centenarian age, thus had a residual lifespan of 15 years and over, had reported a particularly high life satisfaction at age 85. More specifically, out of this small subgroup of sixteen participants, only two participants reported life satisfaction below the median of 8 points.

Finally, we examined effect sizes of the associations between physical and mental health characteristics and life satisfaction with squared Pearson correlation coefficients using the crude, uncategorised, full range variables. For physical health, the explained variance ranged from 0.1% for comorbidity to 3.6% for functional status. The explained variance of life satisfaction for all physical health variables together was 2.8%. For mental health, the explained variance was 25.9% for depressive symptoms and 13.0% for perceived loneliness, and 28.8% combined. When adjusting for demographic and mental health characteristics, the partially explained variance ranged from 0.0 to 2.0% for the various physical health characteristics. The partially explained variance was 23.1 and 11.1% for depressive symptoms and perceived loneliness respectively when adjusting for demographic and physical health characteristics.

## Discussion

We found that among older people age 85, the association between poor physical health and lower life satisfaction was modest, and disappeared when adjusted to mental health, functional status being the exception. Conversely, poor mental health was strongly related to lower life satisfaction, and this association did not change after adjusting for physical health. This suggests that physical health is hardly relevant for older people’s life satisfaction, whereas differences in mental health could distinguish between those with low and those with high life satisfaction.

Life satisfaction varies between high-income Western countries, but is largely unaffected by age within these countries [21]. This observation alone provides reasonable doubt whether age-related disease and disability have an important role in life satisfaction. Earlier publications using selected samples [9, 22] and younger samples [23] already found a limited association between physical health and life satisfaction. In current study, the population of which consisted of a representative sample of older people, this relationship appeared to be even smaller. This is striking, as data from the general population contain a broader range of health states than those from a clinical sample, and greater variety in determinants typically provides larger associations. A possible explanation for may be that participants in clinical samples consider their health more important or feel more burdened by their symptoms than older people of the general population. It is also possible that the larger effects found in other studies were due to confounding by age, which cannot have occurred in the current age-restricted sample. Another relevant contrast to the current study’s findings, is that conversely life satisfaction is a strong predictor of mortality [24], even in older populations [25]. It is possible that life satisfaction leading to worse long-term health outcomes but not vice versa, although sorting this out would require experimental designs, or at least use of longitudinal data and more complex models.

Our analysis showed that participants who died within the first year of follow-up had not reported a lower than average life satisfaction at age 85. This is not in line with earlier findings of a steep drop in life satisfaction in the last year of life that has been suggested as an explanation why life satisfaction decreases in very advanced age [26]. In contrast, we did not find an association between life satisfaction and residual lifespan. This further argues against the idea that high life satisfaction is a reflection of good physical health as indicated by longevity. We did, however, observe that the very few who eventually became centenarians had on average a higher rating of life satisfaction at age 85. If this was not due to physical health, it is tempting to speculate why their wellbeing was higher.

The weak relationship between physical health characteristics and life satisfaction found in the current study provides an important elaboration to a recent proposition of Steptoe, Deaton & Stone [27] that ‘[s]ubjective wellbeing and health are closely related, and the link could become increasingly important at older ages, if only because the prevalence of chronic illness increases with advancing age.’ Our study suggests that while this may hold true for the young old, it may not be true for the oldest old. The low explained variance indicates that health may be less relevant for the latter group. They may hold a more accepting attitude towards disease and disability. This may especially relevant for life satisfaction, as, contrary to hedonic and eudemonic well-being, life satisfaction is thought to consist of the relationship between one’s current experience and one’s future expectations [28, 29]. Expectations may change at old age as poor physical health becomes the norm and there is little expectation of a definite cure [30].

Considering these findings, we suggest that the large role of depressive symptoms and loneliness in life satisfaction for older individuals and the small significance of physical health can be explained by a theoretical model based on the concept vitality [31]. Combining the strengths of oft-cited theories that explain high life satisfaction at old age [29, 32, 33], the model starts from the observation that life satisfaction at old age remains high in the face of disease and disability and that, as qualitative research suggests, maintaining a high life satisfaction is dependent on older people’s ability to adapt personal goals in such a way that they are realistic and meaningful to the individual [30]. We propose that vitality stands for the capabilities that people have at their disposal for setting and achieving appropriate goals and appreciating the results.

### Limitations

The original study sample can be considered characteristic for the Dutch population at 85 years, as there were no exclusion criteria. However, in the present study, we had to exclude those with severe cognitive impairments as we did not have (reliable) estimates for, amongst others, mental health for these subjects. The data can therefore not be extrapolated to participants with severe cognitive impairment. We did, however, obtain ratings on life satisfaction for almost half of them; these ratings were in the same range as for those individuals who were included in the analysis. Furthermore, since we showed that residual lifespan was not associated with life satisfaction.

The presented association between mental health and life satisfaction could be inflated by shared personality factors, such as neuroticism or by environmental factors, such as stressful life events. However, the focus of the current paper was on the relationship between physical health and life satisfaction, and mental health was merely included to find an explanation for the differences in life satisfaction between older people, since we showed that these were not due to differences in physical health. Therefore, in this paper we did not delineate specific affective disorders, nor did we investigate the nature of their association with life satisfaction (as long as it was not due to confounding by physical health). An inflated association between life satisfaction and mental health due to self-reporting could be suggested, but this is rather unlikely, because functional status was also self-reported but failed to show an association with life satisfaction in the same magnitude as mental health.

A weakness of the current study is that mental health characteristics were measured less extensively than physical health characteristics. However, this makes it all the more striking that the level of life satisfaction was so strongly explained by the few mental health measures that were available.

The objective of this study was to uncover the impact of physical and mental health on life satisfaction at advanced age. We found that in general, older people in the Netherlands were highly satisfied with their life, even in the presence of disease and disability. Poor physical health, including cognitive impairment, was not related to lower life satisfaction, and functional status was only weakly related to lower life satisfaction, especially when depressive symptoms and perceived loneliness were taken into account. In contrast, poor mental health was strongly related to lower life satisfaction, and characteristics of physical health did not alter these relationships.

The findings of the present study relate to international criticism and discussion on the use of Gross Domestic Product as a measure of a society’s ‘success’ and ‘well-being’ [34]. As is now widely argued, the relationship between Gross Domestic Product and well-being is not straightforward, and using these constructs interchangeably can lead to a narrow and biased view on well-being and its determinants. Our somewhat unexpected finding that physical health is hardly related to life satisfaction in the oldest old indicates that a similar warning is in order here.

The present data compel refraining altogether from measures that are composed of external determinants of wellbeing. Instead, well-being should be measured by asking people directly [28]. Improving well-being is a priority in public health [35]. and healthcare professionals and policymakers now increasingly make use of self-reported life satisfaction and its correlates to decide on the development and implementation of clinical interventions for individuals or on policies to improve well-being of the population [34]. In line with this, we believe that the present study contributes an important message: it suggests that more success in optimising life satisfaction for older people will come from interventions that prevent depressive symptoms and loneliness than from those that target physical health.

## Conclusion

Among people aged 85, poor physical health was only modestly associated with lower life satisfaction. This association lost significance when adjusted for mental health for all physical health indicators but functional status. At the same time, we found a strong association between poor mental health and lower life satisfaction, and this association did not lose significance by adjusting for physical health. We conclude that at advanced age, physical health is hardly relevant for older people’s life satisfaction. Mental health, however, separates those with low from those with high life satisfaction. Other studies have showed before that mental health has a stronger association with life satisfaction than physical health, but the current study is the first to show that except for functional status, physical health has no unique association with life satisfaction. This is important as it indicates that physical decline may not be a relevant driver of lower life satisfaction at old age, especially when mental health is good.
